# The teaching of management of the pulp in primary molars across Europe

**DOI:** 10.1007/s40368-017-0288-6

**Published:** 2017-05-10

**Authors:** J. Monteiro, A. Ní Chaollaí, M. Duggal

**Affiliations:** 1 Department Paediatric Dentistry, Leeds School of Dentistry, Clarendon Way, Leeds, LS2 9LU England, UK; 2 Private Practice Limited to Paediatric Dentistry, Dublin, Ireland

**Keywords:** Primary pulp therapy, Pulpotomy, Indirect pulp capping, Ferric sulphate, Mineral trioxide aggregate, Formocresol

## Abstract

**Aim:**

To determine which methods of primary pulp therapy are currently being taught in European dental schools.

**Methods:**

An online survey tool was employed to send questionnaires to paediatric dentistry departments of 202 European dental schools. Email addresses were obtained from the European Academy of Paediatric Dentistry and questionnaires were sent to one member of each department in December 2014. The survey included questions on treatment choices and clinical scenarios where respondents indicated how they would advise students to proceed, given a particular case.

**Results:**

Fifty-one responses from 22 different countries were obtained. Eleven schools reported that they taught only undergraduate students, 4 only postgraduates and 36 taught both. Forty-three schools taught indirect pulp capping, the most popular material being calcium hydroxide; 26 taught direct pulp capping, mostly using mineral trioxide aggregate (MTA). Teaching of pulpotomy was widespread across Europe, with MTA being the most popular material, taught in 37 schools, followed by ferric sulphate, in 29. Formocresol, however, was still being taught in 12 dental schools. Responses to the clinical scenarios were mostly in accordance with previously selected choices.

**Conclusions:**

This study had a representative sample, showing a wide variation in primary pulp therapies taught in Europe. Comparison with previous studies shows new trends in taught therapies, possibly driven by recent research in primary pulp management and the development of new materials.

## Introduction

As childhood caries remains a significant oral health issue in Europe, the need for high quality, evidence-based pulp therapy is still necessary and important today. With dmft in 5 year-olds as high as 3.7 in Turkey, 0.8 in England and 1.8 in Germany (6 year-olds), European dental schools must be proficient in teaching the best available techniques to dental students (Gökalp et al. 2010; Public Health England 2015; Santamaria et al. 2015).

During the last decade, developments in the understanding of primary pulp structure, inflammation, healing processes and concerns of the toxicity of dental materials, have led to significant changes in therapeutic choices (International Agency for Research on Cancer 2004). Existing guidelines such as the British Society of Paediatric Dentistry and the American Academy of Pediatric Dentistry have an important role in guiding clinicians through the evidence and advising on adequate management (Rodd et al. 2006; AAPD 2014). Delivery of primary pulp therapy, however, is not without controversy as no single therapy is applicable to all clinical situations. Moreover, recent Cochrane reviews on pulp therapy support this variation. A Cochrane review (Nadin et al. 2003) reported no superiority of one pulp therapy in comparison with others, but it should be remembered that only randomised controlled trials are eligible for inclusion in a Cochrane review, of which there are very few in the relevant paediatric dental literature. However, Smaïl-Faugeron et al. 2014 concluded that although no material had been proven to be superior, there was a tendency for mineral trioxide aggregate (MTA) to have performed better than other pulp therapy products.

A survey initially developed by Primosch et al. (1997) investigated methods of primary pulp therapy taught in pre-doctoral dental programmes in the USA. The authors highlighted the lack of consensus in the management of primary pulp tissue. Subsequent studies in the USA and Brazil shared the same finding (Dunston and Coll 2008; Bergoli et al. 2010).

The present authors have previously conducted a preliminary investigation in the United Kingdom and the Republic of Ireland and reported that all respondents taught the technique of vital pulpotomy, 92% of which used ferric sulphate. There was however, no uniformity regarding pulpectomy, indirect and direct pulp capping. Formocresol and MTA were taught by a minority of respondents. In order to further assess this area of paediatric dental education, and explore the variation between countries, regions and paediatric departments, it was decided to carry out a pan-European survey of the methods of primary pulp therapy currently taught in dental schools.

## Methods

One member of staff was identified as a contact person in each paediatric dentistry department within 202 dental schools located in 36 European countries. Anonymous questionnaires were sent using an online survey tool (sogosurvey^®^) using email addresses that were obtained from the European Academy of Paediatric Dentistry. The survey used was that previously used in a pilot study in Irish and British dental schools (Ní Chaollaí et al. 2009) and was closely adapted from that of Primosch et al. (1997). Feedback received from the pilot study led to adjustments in wording and the number of questions.

The questionnaire was divided into two parts. Firstly respondents were asked about techniques and treatment modalities of choice. In the second part clinical scenarios were given where respondents were asked how they would instruct their students to proceed in those clinical situations. The survey included 23 multiple-choice questions, with the possibility to select ‘other’ and add free text. The number of selected options was unlimited and where more than one option was selected, all answers were included in the analysis.

An email was initially sent to each staff member in December 2014, including an invitation for participation, brief instructions and a link to the web-based survey. The link expired following completion of the questionnaire, eliminating the possibility of repeat answers by the same individual. Two weeks later, an email was sent to all recipients asking them to respond to the survey, if they had not already done so.

## Results

Of the 202 schools contacted, 51 completed the survey from 22 European countries. The majority of schools taught undergraduate and postgraduate students (36), with 11 teaching undergraduate students only and 4 postgraduate students exclusively.

### Part 1: taught techniques and materials

#### Teaching of indirect pulp capping of primary teeth

The majority of respondents answered that they taught indirect pulp capping (43). When asked about the dental materials taught, the responses were: calcium hydroxide (34), glass ionomer cements (20) and zinc oxide eugenol (11). In the free text space five respondents reported that they taught the use of other materials such as MTA, Biodentine™ or TheraCal LC^®^ (Table 1).

**Table 1:** Types of medicaments taught in European dental schools for direct and indirect primary pulp therapy procedures

Indirect pulp therapy	
Medicaments taught for indirect pulp therapy (n = 51)	
Indirect pulp capping was used	43
Calcium hydroxide	34
Glass ionomer cement	20
Zinc oxide eugenol	11
Direct pulp capping	
Medicaments taught for direct pulp therapy (n = 26)	
Calcium hydroxide	22
Mineral trioxide aggregate (MTA)	16

#### Teaching of direct pulp capping of primary teeth

Twenty-six respondents replied that they taught direct pulp capping, mostly using MTA (22) or calcium hydroxide (16). Other materials selected were: glass ionomer cement (4), Total etch™ (4) and Ledermix^®^ (2). Three respondents added Biodentine™ using the free text option. Other respondents reported that this choice was dependent on whether there was a traumatic or carious pulp exposure and whether rubber dam was in use (Table 1).

#### Teaching of pulpotomy in primary teeth

The materials of choice for pulpotomies of primary teeth varied significantly and included: MTA (37), ferric sulphate (29) for the majority of respondents and calcium hydroxide (18), formocresol (12), laser (7) and other materials (10) for the remainder. Respondents who selected other materials specified zinc oxide eugenol, Vitapex^®^, Biodentine™ and Pulpotec^®^. Where more than one material of choice was given, respondents were asked to indicate which medicament was their first choice and the reasons for using an alternative. The majority of respondents considered MTA to be their first choice, followed by ferric sulphate and lastly formocresol. The most common reasons for these choices included success rates, cost and availability of materials. Only 7 respondents were considering changing to a different material, 5 of which to MTA. Interestingly, one respondent replied that their school rarely taught pulpotomy of primary molars due to an increased use of the Hall technique with a preformed metal crown (PMC) (Table 2).

**Table 2:** Details of pulpotomy practices for primary teeth taught in European dental schools

Pulpotomy (n = 51)	
Medicaments taught for vital pulpotomy	
Ferric sulphate	29
Formocresol	12
Mineral trioxide aggregate (MTA)	37
Calcium hydroxide	18
Lasers	7
Other/free text	10
Material placed over pulp following pulpotomy	
Zinc oxide and eugenol cement	29
Reinforced glass ionomer	17
Other/free text	19
Restoration taught following pulpotomy	
Preformed metal crown	34
Composite resin	32
Amalgam	7
Glass ionomer cement	17
Other/specify why	20

When asked which material was taught as appropriate to be applied over a pulp, 29 opted for zinc oxide eugenol and 17 for glass ionomer cement. Nineteen respondents taught the use of other materials, including Biodentine™, MTA, calcium hydroxide and guta percha (Table 2).

Respondents were asked which restorations they taught following pulpotomy of primary molars, with preformed metal crowns (PMC) and composite resin being the most popular (34 and 32 respectively). A minority of respondents taught glass ionomer cement (17) or amalgam (7). In the free text option the majority of respondents discussed that their decision was dependent on the extent of the cavity and whether it was a single or multi-surface restoration (Table 2).

Following placement of the final restoration, 16 respondents instructed their students to take a post-operative radiograph immediately. The remainder taught post-operative radiographs at 6 months (21) or 12 months (12) following pulpotomy. Finally, 19 respondents taught students to take radiographs at a time interval determined by the child’s caries-risk.

### Part 2: clinical scenarios

**Fig. 1:**
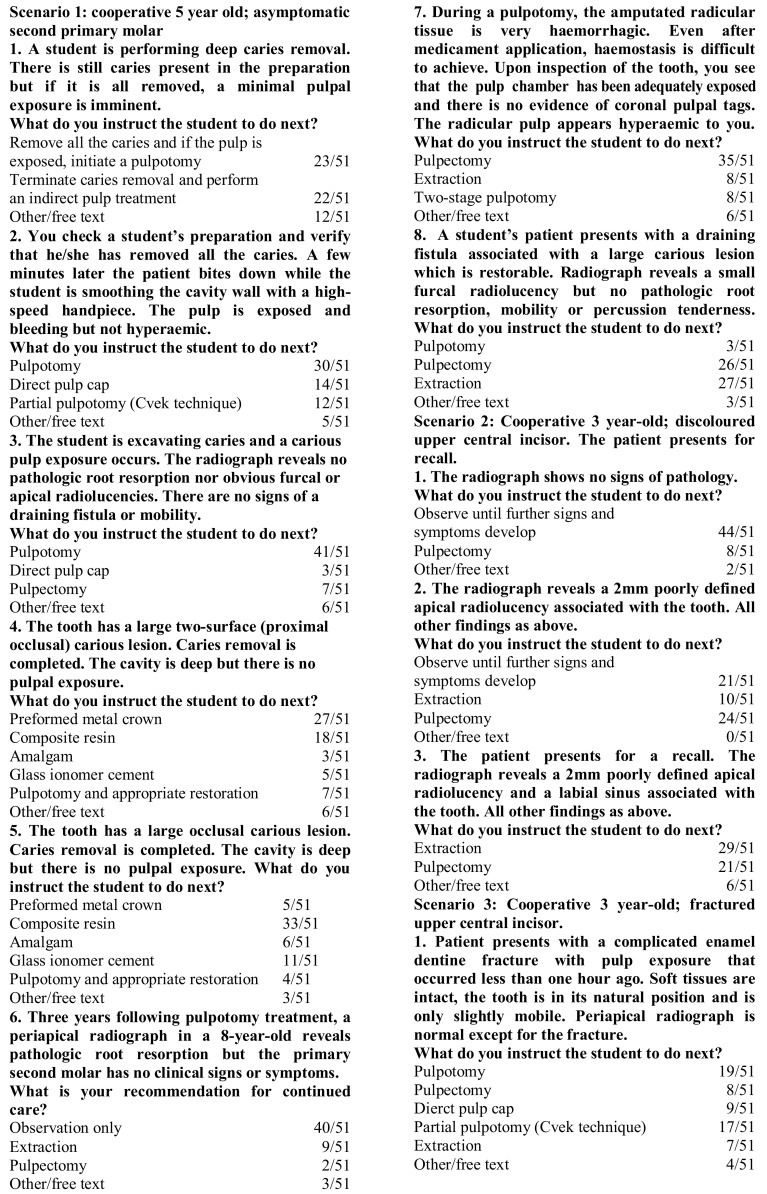
Clinical scenario questionnaires and responses referring to a healthy child in three scenarios (after Primosch et al. 1997)

In this section respondents were asked to select options that would more closely resemble how they would instruct their students, given three case scenarios. The questions and answers are presented in Fig. 1. When more than one option was selected respondents were asked to discuss their choice of several answers. Similarly, when ‘other’ was selected, respondents were asked to explain their answer.

#### Scenario 1

Case 1 referred to a cooperative 5 year old with an asymptomatic second primary molar. During deep caries removal, if the tooth was still carious and a pulp exposure was likely, respondents were mostly divided between teaching the use of a pulpotomy (23) or an indirect pulp cap (22). However, 12 respondents provided alternative answers, mostly including step-wise excavation or discussed that their answer was dependent on whether caries was occlusal or approximal. If the patient were to accidentally bite down on a rotating bur exposing the pulp in a clean cavity (non-hyperaemic pulp), the majority would recommend performing a pulpotomy (30). Respondents who selected ‘free text’ specified that their choice would depend on a child’s medical history and the use of rubber dam. Responses were unequivocal in the scenario of carious pulp exposure, with most respondents instructing their students to undertake a pulpotomy (41). Six respondents included additional text discussing that their choice would depend on a child’s cooperation or that they might proceed differently to how they would teach their students. 

When discussing the restoration of choice, on a deep two-surface cavity, respondents would mostly teach using a PMC (27) or a composite resin (18). Free text reiterated the respondent’s choices but provided no alternatives. In similar circumstances but with an occlusal cavity, the material of choice was mostly composite resin (33). Free text alternatives included compomer. Following a pulpotomy, in the presence of pathologic root resorption and absence of clinical signs or symptoms, the vast majority of respondents would monitor the patient (40) with free text provided by three respondents, discussing that treatment was dependent on parental preferences and size of radiolucency. If the pulp was thought to be hyperaemic during a pulpotomy, 35 respondents would advise a pulpectomy, with only eight instructing the students to extract the tooth. Free text answers added that this decision would be dependent on the degree of root resorption. In contrast, if there was a draining fistula associated with the tooth, respondents were divided between pulpectomy and extraction (26 and 27, respectively).

#### Scenario 2

Scenario 2 related to a cooperative 3-year-old presenting for recall with a discoloured maxillary central incisor and no signs of radiographic pathology. In this case the majority of respondents would monitor the case until further symptoms and signs develop (44). There was less agreement if a 2 mm apical radiolucency was present, as respondents would either observe (21) or teach a pulpectomy (24), with 10 respondents advocating extraction. If a labial sinus was also present the majority would opt for extraction (29) or pulpectomy (21). In this scenario free text comments added by respondents were limited to explanation of the materials of choice.

#### Scenario 3

Scenario 3 related to a cooperative 3-year-old with a complicated crown fracture of a maxillary central incisor. Respondents would mostly advise pulpotomy (17) or partial pulpotomy (19). Free text informed that this decision was dependent on the size of the exposure.

## Discussion

The present study obtained a representative sample from across Europe, that included 51 dental schools, from 22 countries. Despite the highly representative sample, the authors speculate on the exclusive use of the English language and unknown changes to email addresses as possible factors for a lower than expected participation rate, when compared to the pilot study (87.5%) (Ní Chaollaí et al. 2009).

The widespread geographic location of the sample may have accounted for a greater variation in responses when compared to the pilot study, limited to Ireland and the United Kingdom (Ní Chaollaí et al. 2009). In the present survey, responses always included all choices presented and the free text option was frequently used to discuss different approaches to those contemplated by the authors. Amongst these, new therapies such as Biodentine™ emerged in this survey as a new European trend. It became clear that the wide range of techniques and materials taught was an indicator of the lack of consensus on the most appropriate therapies for primary pulp management. However, as new therapies are being used and, most importantly, taught, well-conducted research is of utmost importance, hence the need for further randomised controlled trials to evaluate the various techniques.

Nevertheless, the present survey found that most respondents taught evidence-based techniques, supported by current national and international guidelines (Rodd et al. 2006; AAPD 2014). Furthermore, teaching of MTA was widespread across Europe, undoubtedly reflecting the high success rates obtained from studies over the last few decades (Holan et al. 2005; Ng and Messer 2008). Cost, however, remains a prohibiting barrier in many countries.

Conversely, formocresol and formocresol-containing materials were still being taught to 23.5% of European students, which was very similar to the earlier pilot study conducted by the present authors (Ní Chaollaí et al. 2009). This is an interesting finding, in the light of formocresol’s classification as carcinogenic to humans by the International Agency for Cancer Research (IACR), over a decade ago (IARC 2004). Respondents, regrettably, did not discuss the reasons behind this choice. As American authors found a decline in formocresol’s teaching on repeat surveys, one may speculate that this trend might be replicated in future surveys of the European population (Dunston and Coll 2008).

A good coronal seal is crucial for the success of any pulp therapy. A number of studies have shown increased survival of PMCs in comparison to other materials (Roberts et al. 2005). A recent update of a Cochrane review concluded that PMCs on carious teeth or following pulpotomy are likely to reduce the risk of major failures in the long term (Innes et al. 2015). In the present survey 34 respondents instructed the use of PMC following a pulpotomy and a small number indicated that their decision depended on the extent of the cavity. Similar reasoning was supportive of an increased use of PMCs on two-surface caries lesions when compared to occlusal cavities, where composite was the material of choice. Interestingly, the choice of material was not without controversy which was a consequence of the recent Minamata Convention, in October 2013, where over 100 countries agreed to phase out the use of amalgam until 2017, with Norway leading the way in Europe. Once again this might introduce changing trends in future surveys (FDI World Dental Federation 2014).

Although a considerable number of schools would teach direct pulp capping, this did not translate to the management of hypothetical scenarios, where direct pulp capping was seldom selected. This is in accordance with the current literature, which generally discourages the use of direct pulp capping on primary teeth due to the low success rates (Rodd et al. 2006).

Teaching of indirect pulp capping seems to have increased and was widespread across Europe. This was clear in the management of the clinical scenarios, where almost equal numbers of respondents would manage an imminent exposure with either pulpotomy or indirect pulp capping. Furthermore, in the free text option, several respondents discussed teaching the Hall technique, with one respondent reporting that this was the treatment of choice in their school with an accompanying reduction in the use of pulpotomy. Indirect pulp capping, however, still remained a subject of controversy, arguably due to the challenges in determining pulpal status in young and uncooperative children (Fuks et al. 2016). Although it is well known that primary pulp tissue behaves in a number of ways to different caries sites, the clinical and histological diagnosis of irreversible pulpitis has yet to be defined (Duggal et al. 2002; Kassa et al. 2009). Being the subject of much of the current research in this field, the roles of indirect pulp capping and of the Hall technique are expected to become more defined in the near future (Innes et al. 2011, 2013; Santamaria et al. 2015).

## Conclusions

This survey found a wide variation of taught techniques, clearly illustrating that the management of the primary pulp remains an evolving field. This is of particular interest at a time where mobility of dentists across Europe seems to be increasing. Students taught in one country during their undergraduate or postgraduate degrees may become established in a different country, making regional differences in teaching increasingly relevant to their practice.
